# Three-Dimensional Virtual Bone Bank System Workflow for Structural Bone Allograft Selection: A Technical Report

**DOI:** 10.1155/2013/524395

**Published:** 2013-04-09

**Authors:** Lucas Eduardo Ritacco, German Luis Farfalli, Federico Edgardo Milano, Miguel Angel Ayerza, Domingo Luis Muscolo, Luis Aponte-Tinao

**Affiliations:** ^1^Virtual Planning and Navigation Unit, Department of Health Informatics, Italian Hospital of Buenos Aires, 1199 Buenos Aires, Argentina; ^2^Institute of Orthopedics “Carlos E. Ottolenghi”, Italian Hospital of Buenos Aires, Potosí 4247, 1199 Buenos Aires, Argentina

## Abstract

Structural bone allograft has been used in bone defect reconstruction during the last fifty years with acceptable results. However, allograft selection methods were based on 2-dimensional templates using X-rays. Thanks to preoperative planning platforms, three-dimensional (3D) CT-derived bone models were used to define size and shape comparison between host and donor. The purpose of this study was to describe the workflow of this virtual technique in order to explain how to choose the best allograft using a virtual bone bank system. We measured all bones in a 3D virtual environment determining the best match. The use of a virtual bone bank system has allowed optimizing the allograft selection in a bone bank, providing more information to the surgeons before surgery. In conclusion, 3D preoperative planning in a virtual environment for allograft selection is an important and helpful tool in order to achieve a good match between host and donor.

## 1. Introduction

The uses of bone allograft after bone tumor resection have been described with acceptable results in osteoarticular, transepiphyseal, and intercalary reconstructions [1–4]. 

Selection of the closest anatomical match between the host and the donor is crucial in order to obtain adequate joint stability, alignment, appropriate wound closure, and minor degenerative changes of the articular surface in osteoarticular allograft.

Since 1950, bone allograft selection according to size and shape for limb reconstruction was made by comparing X-ray images between donors and patient [5]. This method had inaccuracies between the X-ray magnification scale and real bone, altering the final selection [6]. In the 1970s, CT scanner allowed to refine these inaccuracies taking into account one image slice in two dimensions [7]. The previous two decades have seen an increase in the use of virtual scenarios and informatics developments for preoperative planning [8–10]. Three-dimensional patient-specific anatomical models can be constructed from medical image data.

We described a virtual technique capable of selecting a suitable allograft according to size and shape through a three-dimensional virtual model. 

The aim of this paper was to describe the workflow of this technique in different cases in order to explain how to choose the best allograft using a virtual bone bank system.

## 2. Material and Methods

Three-dimensional (3D) virtual bone models from host and donor were obtained following these steps: *image acquisition*, *image segmentation,* and *3D bone reconstruction*, described in detail below.

Once this workflow was completely defined, we were capable of measuring each bone in a virtual environment and establishing 3D comparisons between host and donor to determine the best match. 

All images were acquired using a CT scanner (Mutislice 64, Aquilion, Toshiba Medical Systems, Otawara, Japan). Magnified slices with 0.5 mm thickness were obtained using a soft tissue standard filter, a matrix of 512 × 512 pixels, and stored in Digital Imaging and Communication in Medicine (*DICOM*) format.

In order to establish comparable measurements, image acquisition protocols should be equal in host and donor. Image magnification process is an important step to obtain images in high definition. Thus, we suggest magnifying the image as much as possible.

**Figure 1:**
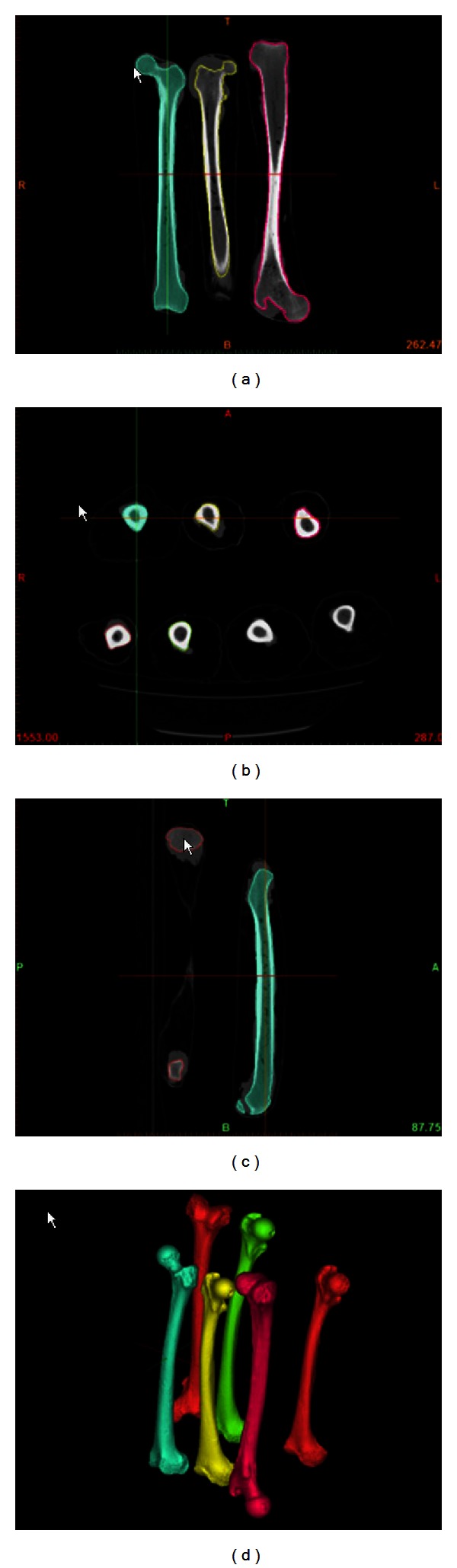
(a)–(c) Image segmentation. (d) Bone allograft was 3D reconstructed.

Image segmentation is a process which consists in changing the representation of a DICOM image into an image that is easier to analyze. We used a specialized software for the segmentation tasks (Mimics software, Leuven, Belgium). Through this process, an operator assigns a color to every pixel in an image such that pixels with the same intensity define a separate structure: for example, cortical and trabecular bone. In our case, the bone after the segmentation process was isolated from the other tissues and structures such as muscle, fat, skin, ice, and metal table of CT scanner (Figure 1).

The result of image segmentation is a set of segments or a set of contours extracted from the image that collectively cover the entire 3D bone model. Each of the pixels in a region is similar according to intensity. The resulting contours after image segmentation were used to create 3D reconstructions with the help of interpolation algorithms. In this manner, a three-dimensional bone model was created (Figure 1). The segmentation process of a whole large bone takes a mean of 10 hours. 

Take into account that the “contrast” in the CT for image segmentation is the calcium density. In oncologic patients, pain and lack of mobility lead to low calcium density. In consequence, the cortical and trabecular bones are replaced by the tumor action, erasing the anatomical shape and recognizable landmarks. In this way, 3D tumoral bone models appear to be incomplete. 

Hereby, exploiting the symmetry of the human body [11, 12], we create a 3D mirror model from the patient's healthy side.

**Figure 2:**
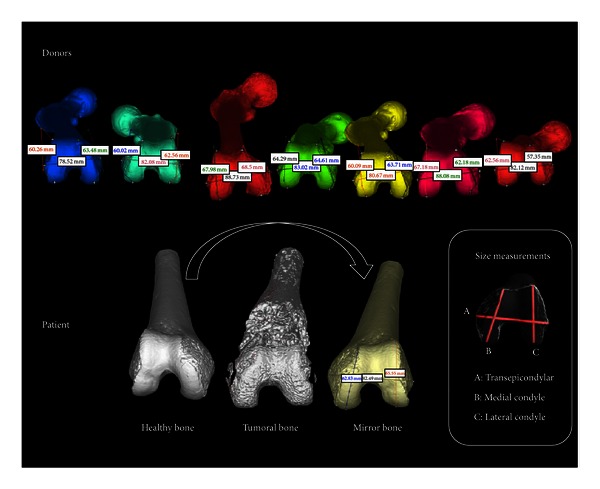
Donors were measured using ABC measurements. The healthy bone of the patient was mirrored and then measured with ABC measures in order to compare the best match according to the sizes.

In order to select the best size, six anatomical landmarks were defined determining three principal measures from the 3D mirror model: A is transepicondylar, B is medial anterior-posterior condyle, and C is lateral anterior-posterior condyle (Figure 2). Once the whole bank was measured we created a table with all the ABC extents. First we search, as a screening step, ABC donors closest to ABC host. Next, in order to compare and select the best shape, 3D mirror model is overlapped with the available donors. Before comparing 3D shapes, all 3D bones were positioned in the same coordinate system. This process is called 3D registration.

Using a point cloud model of surfaces, it was possible to obtain a numeric value (a mean) that reflects the goodness of the match [12].

Distances between host and donor were illustrated in a colorimetric mapping. 

**Figure 3:**
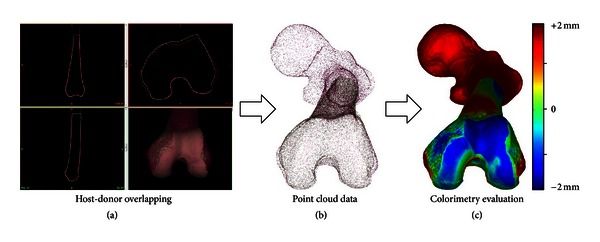
(a) Host and donor were overlapped in a virtual platform in order to compare the best match according to the shapes. (b) 3D models were exported to point cloud data. (c) A colorimetry evaluation was applied comparing host and donor surfaces.

This tool allowed us to determine which is the most similar area with a color scale (Figure 3).

**Figure 4:**
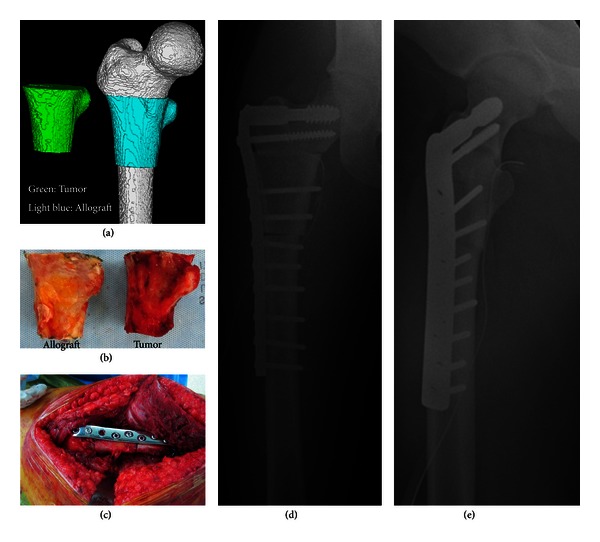
(a) Bone allograft was selected according to the shapes comparison between host and donor. The original tumor diagnosis was a chondrosarcoma. (b) Allograft was selected and tumor was resected. (c) Allograft was fixed in the patient through a plate and screws. (d) and (e) Postoperative X-rays.

Since it is not an easy task to determine natural landmarks in transepiphyseal and diaphyseal allografts, only it is possible to determine a match by overlapping the host with available donors (Figure 4).

## 3. Discussion

Paul et al. in their study [11] explored the use of 2-dimensional template comparison for allograft matching. However, the cited study also describes a 3D registration method and states that the 2-dimensional template comparison is ineffective. 

The correspondence between the 3D models and the real bone depends heavily on CT scanner, segmentation, and interpolation software [11].

Published studies on three-dimensional preoperative planning using virtual environments for allograft selection have reported benefits in pelvis and femur [11–14]. The use of this technology allowed optimizing the allograft selection in a bone bank, providing three-dimensional visual information to the surgeon before surgery is executed.

As well, if the host has to be compared against multiple available donors, the process would be very time consuming if it were to be performed manually [14]. Actually, the algorithms described in the cited articles were capable of automatically choosing the best allograft according to the size and shape criteria. We also have already published acceptable results applying an automatic method to select the best match [15, 16]. 

Although we know that anatomical matching is only one of multiple factors that could affect the outcome of an allograft reconstruction, poor matching between host and donor can alter joint kinematics and load distribution, leading to bone resorption and joint degeneration [17, 18]. Pathological studies showed that allografts retrieved from patients with a nonsimilar joint had earlier and more advanced degenerative changes in the articular cartilage than did allografts retrieved from patients with a stable joint [17, 19]. 

## 4. Conclusion 

We consider that a three-dimensional preoperative planning in a virtual environment for allograft selection is an important and helpful tool in order to achieve a good match between host and donor. Currently, we are following these patients to assess their limb function at several postoperative intervals.
